# *In vivo* protective effect of adipsin-deficiency on spontaneous knee osteoarthritis in aging mice

**DOI:** 10.18632/aging.102784

**Published:** 2020-02-01

**Authors:** Frédéric Paré, Ginette Tardif, Hassan Fahmi, Yassine Ouhaddi, Jean-Pierre Pelletier, Johanne Martel-Pelletier

**Affiliations:** 1 Osteoarthritis Research Unit, University of Montreal Hospital Research Centre (CRCHUM), Montréal, Québec, Canada

**Keywords:** osteoarthritis, adipsin, aging, FGF-21

## Abstract

The adipokine adipsin is an emerging mediator of human osteoarthritis (OA) progression. Here, we investigated its in vivo role in the development of spontaneous OA in aging mice. We compared articular knee joint morphology, histology in knee cartilage, synovial membrane, subchondral bone, meniscus, and anterior cruciate ligament (ACL); and chondrogenesis in the ACL from adipsin-deficient (*Df*^-/-^) and wild-type (*Df*^+/+^) 20-week- and 20-month-old mice. Serum levels of a panel of adipokines, inflammatory factors, and metalloproteases known to be implicated in OA were investigated. Data first revealed that the early manifestation of OA appeared in the ACL of 20-week-old mice, progressing to severe alterations in the 20 month-old wild-type mice. Further results demonstrated that adipsin-deficiency protected the articular tissues from spontaneous OA progression and triggered significantly higher serum levels of the adipokines adiponectin and FGF-21 while lowering levels of the inflammatory factor interleukin 6 (IL-6) in both young and old mice. This work further underlines the clinical relevance of adipsin as a novel therapeutic approach of human OA. Moreover, this study shows the potential beneficial effect of the adipokine FGF-21 against OA, and provides support for this factor to be a new biomarker and/or target of primary OA therapeutic avenues.

## INTRODUCTION

Age is a major risk factor for the development of the most common musculoskeletal disease, osteoarthritis (OA). Primary OA affects more than half of the world’s population aged 65 and older. This disease is a leading cause of disability worldwide and one of the most common chronic illnesses, accounting for 40-60% of patients with degenerative diseases [1]. OA can affect many articulations, but it is commonly localized in the weight-bearing joints and most frequently occurs in the knee. This debilitating disease results in a progressive alteration of all the joint tissue structures [2]. Although aging does not necessarily cause OA, age-related changes coupled with other risk factors could accelerate the development of its pathological process [3, 4].

Currently, there is no cure for OA nor way to prevent the disease’s progression. Available treatments are effective only in relieving symptoms, and in older adults their use could induce major adverse events and even mortality [5]. An important difficulty in developing disease-modifying OA drugs (DMOADs) is identifying patients from an early stage of the disease. Further efforts are needed to move toward a better understanding of the factors and joint tissues implicated early in this disease process and to open up novel therapeutic avenues.

As the evolution of joint structural alterations in human OA occurs over an extended period, animals offer the advantage of manifesting this disease development within a shorter time than humans, enabling investigation of this disease in a timely fashion and of a more global scope. In animals, OA models have been mainly induced following surgery, with the mouse model of destabilization of the medial meniscus (DMM) of the knee being the most used one [6]. Although informative as to the response to a traumatic surgery, it does not answer several questions pertaining to the events leading to the development in primary OA.

A number of genes and factors have been implicated in the OA [7–9] and aging [10, 11] processes. Several pro-inflammatory cytokines have been found to play a role in each process; factors such as interleukin (IL)-6, tumor necrosis factor alpha (TNF-α), and C-reactive protein (CRP) are significant contributors not only in elderly individuals [12–15], but their importance is also well-documented in OA development [7, 16–19]. In addition, the production of adipokines and inflammatory factors by adipose tissues is also established [20–22]. Adipokines are molecules that regulate energy metabolism as well as the production of inflammatory factors [23–25], and the levels of certain adipokines have been found deregulated in the above-mentioned conditions [10, 26–30]. The adipokine adipsin is an emerging mediator of human OA progression [26–28]. It is mainly produced by the adipose tissue and is an integral component of the alternative complement pathway [31]. Data revealed that adipsin-deficiency delayed OA progression in a DMM-induced OA mouse model which appeared to result, at least in part, from a decreased activity of the alternative complement pathway [27]. Importantly, the aberrant activation of the alternative complement pathway has been implicated not only in OA and the aging process, but also in a number of other age-related diseases including diabetes, age-related macular degeneration, and Alzheimer’s [32–36].

We therefore hypothesize that adipsin-deficiency will prevent the spontaneous development of OA in mice.

## RESULTS

### Wild-type and adipsin-deficient mouse model

As mentioned in the Methods section Histology/ Histomorphometry, about half of the wild-type (*Df*^+/+^) aging mice demonstrated severe knee lesions with a high cartilage degradation score and were named *Df*^+/+^(H); the other *Df*^+/+^mice had a lower score and were named *Df*^+/+^(L). None of the adipsin-deficient mice (*Df*^-/-^) presented a high cartilage degradation score. Further analysis with the Fisher’s exact test revealed that the phenotype of the adipsin-deficient mice was not due to chance (p=0.015).

### Micro-CT (μCT) of the knee joint

**Figure 1:**
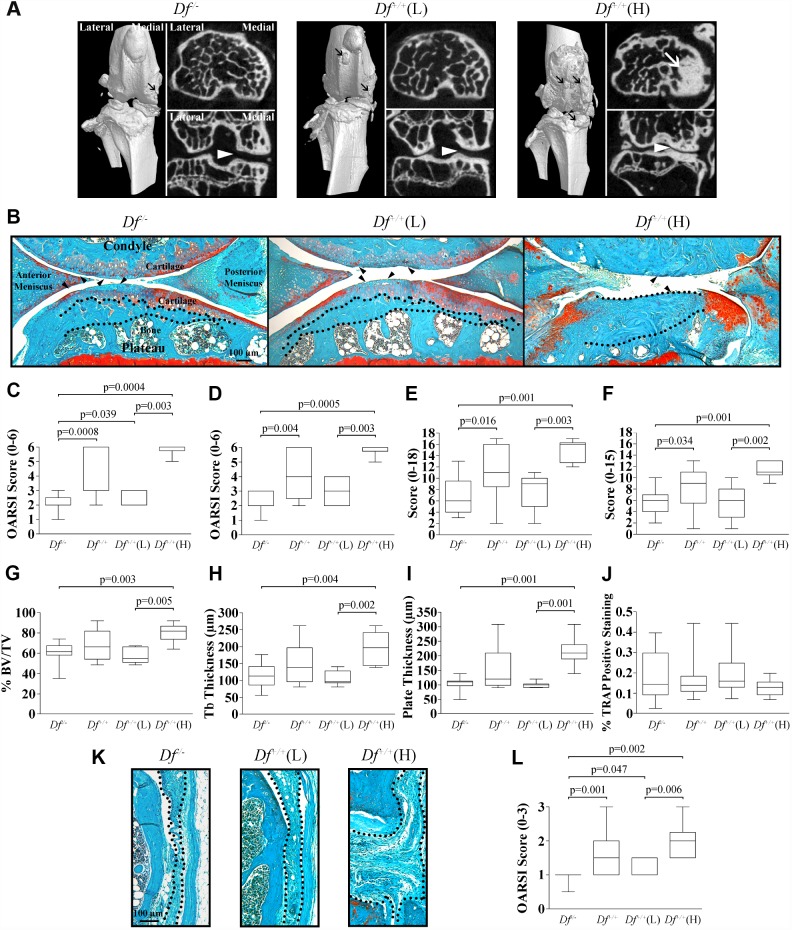
**Micro-CT (μCT) and histology of joint tissues of 20-month-old mice.** (**A**) Knee joint μCT of adipsin-deficient (*Df*^-/-^), and the wild-type *Df*^+/+^(L) and (H) mice. Representative 3-dimensional reconstructions of the joint and axial and coronal images of the subchondral bone compartment. Black arrows indicate osteophytes, white arrows sclerosis and the white arrowheads joint space in the medial compartment. (**B**) Photomicrographs of representative histological sections of joint tissues: cartilage, meniscus and subchondral bone. The dotted lines delineate subchondral plate thickness and the black arrowheads cartilage alterations. Bar = 100 μm. Original magnification X63. (**C**–**J**) Representative box plots of the Osteoarthritis Research Society International (OARSI) score of the (**C**) medial tibial plateau, and (**D**) medial femoral condyle; the (**E**) anterior and (**F**) posterior menisci; the subchondral bone assessment of the (**G**) percentage of bone (% bone volume [BV]/total volume [TV]), (**H**) trabecular (tb) thickness, (**I**) plate thickness, and (**J**) tartrate resistant acid phosphatase (TRAP) assay. (**K**) Photomicrographs of representative histological sections of the synovial membrane and (**L**) box plot of the OARSI score of the synovial membrane. In (**K**) the dotted lines delineate synovial membrane thickness. Bar = 100 μm. Original magnification X100. For each box plot, values are the median and interquartile range of *Df*^-/-^ (n=13), *Df*^+/+^ (n=13), *Df*^+/+^(L) (n=7) and *Df*^+/+^(H) (n=6). p values were determined by the Mann-Whitney test and only significant values are shown.

As illustrated in Figure 1A, the μCT analysis of the joints of the 20-month-old mice showed that compared to the adipsin-deficient (*Df*^-/-^) and the wild-type (*Df*^+/+^) (L), the *Df*^+/+^(H) knees presented very severe OA knee alterations with a high number of osteophytes, large subchondral bone sclerosis, and a marked narrowing of the joint space in the medial compartment. By using this technology, no changes between the *Df*^-/-^ and the *Df*^+/+^(L) mice could be identified.

### Adipsin-deficient mice are protected from spontaneous OA progression

The impact of the lack of adipsin production on the evolution of spontaneous OA occurring in aging mice was evaluated. Figure 1B–1L compares the joint tissues of the 20-month-old *Df*^-/-^ with those from *Df*^+/+^, *Df*^+/+^(L) and (H) mice. Tissues from the 20-week-old mice are not presented, as the comparison of the articular tissues (cartilage, subchondral bone, and synovial membrane) between *Df*^-/-^ and *Df*^+/+^ mice has already been reported, showing no histological differences nor early signs of OA process in these tissues [27].

### Cartilage

Figure 1B illustrates cartilage degradation in both medial tibial plateau and femoral condyle of the 20-month-old in which the *Df*^+/+^ mice demonstrated a higher loss of cartilage integrity, and a high Osteoarthritis Research Society International (OARSI) score [37], including decreased Safranin-*O* staining, altered cellularity, increased thinning of the cartilage and fibrillation. The more severe degradation occurred in the cartilage of the *Df*^+/+^(H) mice (both for the femoral condyle and tibial plateau), as this tissue was almost completely eroded. Compared to the *Df*^+/+^(H) mice, the cartilage from *Df*^+/+^(L) and *Df*^-/-^ had a better cartilage structure and less degradation. The histological score of the *Df*^-/-^ mice was significantly lower compared to those of *Df*^+/+^, *Df*^+/+^(L) and (H) mice for the medial tibial plateau (Figure 1C) and to those of *Df*^+/+^ and *Df*^+/+^(H) for the femoral condyle (Figure 1D). Moreover, significant differences were also seen between *Df*^+/+^(L) and *Df*^+/+^(H) for both compartments.

### Meniscus

The histological examination of the *Df*^-/-^ meniscus revealed a significantly lower level of degradation of both the anterior and posterior compartments compared to those of the *Df*^+/+^ and *Df*^+/+^(H) mice (Figure 1B, 1E, and 1F). No differences in the histological scores were observed between the *Df*^-/-^ and *Df*^+/+^(L) groups, but significance was reached between the *Df*^+/+^(L) and *Df*^+/+^(H) groups for each compartment (Figure 1E, 1F). The *Df*^+/+^(H) meniscus (Figure 1B) presented a high score, in which altered cellularity, decreased matrix Safranin-*O* staining and increased fibrillation in both the anterior and posterior compartments were found.

### Subchondral bone

The analysis of the subchondral bone (Figure 1B, 1G–1I) showed that the adipsin-deficient (*Df*^-/-^) mice had a significant decrease in the percentage of bone, trabecular and plate thickness when compared to the *Df*^+/+^(H) mice, but no significant differences were found with the *Df*^+/+^(L) subgroup. As for the cartilage and meniscus, significantly higher values were obtained for the *Df*^+/+^(H) mice when compared to *Df*^+/+^(L) mice.

The tartrate resistant acid phosphatase (TRAP) assay was performed to monitor osteoclast activation in the subchondral bone. Data showed that only a few TRAP-positive cells were detected in the subchondral bone around the bone marrow. As illustrated at Figure 1J, there was no difference in the TRAP positive staining between groups. One could question the finding for the *Df*^+/+^ (H) mice, however, this data was not surprising since in these mice there were important sclerosis (Figure 1B *Df*^+/+^ (H)) and only few bone marrow remained in which few TRAP-positive cells could be detected.

### Synovial membrane

The *Df*^-/-^ synovial membrane presented a significantly reduced score when compared to those of the *Df*^+/+^, *Df*^+/+^(L) and (H) subgroups (Figure 1K–1L). Again, *Df*^+/+^(H) mice demonstrated a significantly higher level of alterations than the *Df*^+/+^(L) mice. Of note, there is a substantial increase in the synovial membrane thickness and hyperplasia of the lining cells in the *Df*^+/+^(H) mice (Figure 1K).

### Anterior cruciate ligament (ACL)

**Figure 2:**
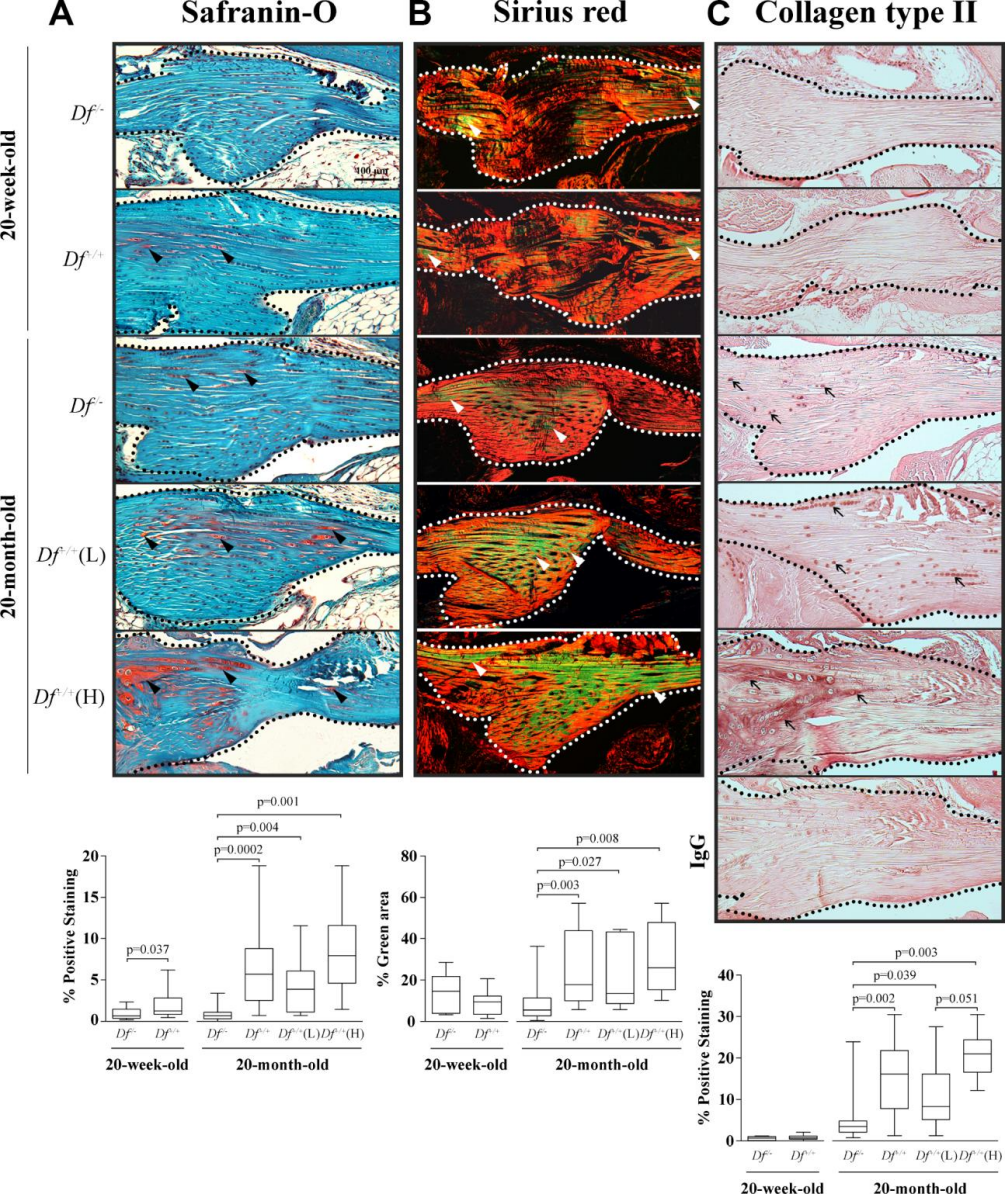
**Histology and type II collagen deposition in the anterior cruciate ligament (ACL).** Photomicrographs of representative images and box plots of 20-week-old adipsin-deficient (*Df*^-/-^) and wild-type (*Df*^+/+^) and 20-month-old *Df*^-/-^, *Df*^+/+^, *Df*^+/+^(L) and (H) mice of (**A**) Safranin-*O* staining, black arrowheads indicate proteoglycans deposition; (**B**) Sirius red staining enabling visualization of the collagen fibers. The green fibers corresponding to altered collagen were quantified over the total area. White arrowheads indicate thin collagen fibers. (**C**) Immunohistochemistry of type II collagen deposition and a negative control (IgG) performed by substitution with a non-specific rabbit IgG. Black arrows indicate positive staining. In (**A**–**C**) dotted lines delineate the core portion of the ACL. Bar in (**A**) = 100 μm. Original magnification X100. Values are the median and interquartile range of *Df*^-/-^ (n=11), *Df*^+/+^ (n=13) for the 20-week-old mice and of *Df*^-/-^ (n=13), *Df*^+/+^ (n=13), *Df*^+/+^ (L) (n=7) and (H) (n=6) for the 20-month-old mice. p values were determined by the Mann-Whitney test. Only significant differences are shown except for those comparing 20-week-old and 20-month-old *Df*^-/-^ (**C**, p= 0.0001) and *Df*^+/+^ (**A**, p= 0.006; **B**, p=0.004; **C**, p< 0.0001) mice.

In the ACL, the earliest changes during the aging process and preceding radiological signs of OA are mucoid degeneration (degradation of collagen and deposition of new glycosaminoglycans), collagen fiber disorganization, and the presence of chondrocyte-like cells and type II collagen deposition [38–41]. We evaluated these parameters in the ACL from 20 week- and 20 month-old *Df*^-/-^ and *Df*^+/+^ mice (Figure 2).

Data showed that in young animals, Safranin-*O* staining level was significantly lower in *Df*^-/-^ compared to *Df*^+/+^ mice (Figure 2A), suggesting that early alteration occurs in the ACL at a young age, at 20-weeks-old. Moreover, data also revealed that adipsin-deficiency protected from such an alteration, as comparison between the younger and older mice revealed that the Safranin-*O* staining level remained stable in the *Df*^-/-^ animals. In the 20-month-old mice, a significantly higher level of Safranin-*O* staining was found in *Df*^+/+^mice compared to *Df*^-/-^ mice, with the level about tripled. In these mice, significantly higher levels were also found for both *Df*^+/+^(L) and (H) subgroups than in *Df*^-/-^.

Collagen organisation data (sirius staining, green color indicating collagen fibers alterations) (Figure 2B) also showed that older adipsin-deficient mice were protected against this collagen alteration. In contrast, in the 20-month-old mice, as observed with Safranin-*O*, the level of staining for the *Df*^+/+^mice about doubled when compared to *Df*^-/-^ mice; this was also true for both *Df*^+/+^(L) and (H) mice. No differences in the adipsin-deficient *Df*^-/-^ mice were found between 20-week- and 20-month-old mice.

Determination of type II collagen deposition (Figure 2C) revealed that this collagen type was virtually absent in the 20-week-old *Df*^-/-^ and *Df*^+/+^mice and increased in the 20-month-old. As with the two other ACL measurements, in the older mice, the adipsin-deficient mice had significantly less type II collagen than *Df*^+/+^ and both *Df*^+/+^(L) and (H) subgroups. Of note, there was also a statistically significant higher level of type II collagen in the *Df*^+/+^(H) mice compared to the *Df*^+/+^(L).

Altogether, data on the ACL indicates that changes occur in this tissue as early as 20-weeks-old and progress with age.

### Impact of adipsin-deficiency on the serum levels of some adipokines/inflammatory factors/proteinase

Considering that the previous results demonstrated that adipsin-deficiency significantly reduced the spontaneous manifestation of OA in several articular tissues, we further evaluated if this was reflected by a change in the serum levels of factors related to both OA and aging. Among the studied factors, the serum levels of granulocyte-macrophage colony-stimulating factor (GM-CSF), vascular endothelial growth factor (VEGF), S100 calcium-binding protein A8 (S100A8), receptor for advanced-glycation-end-products (RAGE), interleukin (IL)-7, IL-10, IL-17 and TNF-α could not be determined accurately, as values were under the detection limit of the assays. Serum levels of hepatocyte growth factor (HGF), monocyte chemoattractant protein (MCP)-1, matrix metalloproteinase (MMP)-8 and S100 calcium-binding protein A9 (S100A9) were detectable, but no significant differences were reached between *Df*^-/-^ and *Df*^+/+^ mice in either the 20-week- or the 20-month-old animals (data not shown).

As presented in Table 1, for the adipokines, significantly higher serum levels were found in the *Df*^-/-^ than *Df*^+/+^ mice for adiponectin and fibroblast growth factor (FGF)-21 in both young and old mice. Leptin had a reverse pattern; when the *Df*^-/-^ was compared to the *Df*^+/+^ mice, its levels were decreased in the young mice, but increased in the older ones. Resistin levels were significantly higher in the young *Df*^-/-^ mice but did not vary in the older mice. Interestingly, for the 20-month-old mice, difference between the *Df*^-/-^ with *Df*^+/+^(L) and (H) mice subgroups revealed that although adiponectin and leptin levels were significantly higher compared to the *Df*^+/+^(L) group, no significant difference was found for the (H) group. For FGF-21, *Df*^-/-^ had significantly higher levels than both *Df*^+/+^(L) and (H) mice subgroups.

**Table 1:** Serum levels of adipokines/inflammatory factors/proteinase.

	**20-week-old mice**		**20-month-old mice**				**Age comparison**	
	***Df^-/-^ (n=11)***	***Df^+/+^* p^‡^ (n=13)**	***Df^-/-^ (n=9)***	***Df^+/+^* p^‡^ (n=11)**	***Df^+/+^*(L) p^†^ (n=7)**	***Df^+/+^*(H) p^††^ (n=4)**	(*Df^-/-^*) p^*^	(*Df^+/+^*) p^**^
Adiponectin (ng/ml)	5088 (4788; 5615)	4632 (4397; 4854) **0.030**	4942 (4601; 5369)	4166 (3562; 4410) **0.005**	3787 (3562; 4206) **0.008**	4410 (3822; 4588) 0.076	0.323	0.132
FGF-21 (pg/ml)	432.1 (359.5; 586.8)	307.8 (142.0; 430.8) **0.032**	1688.1 (901.9; 1697.4)	398.4 (219.5; 823.9) **0.002**	314.0 (195.4; 630.7) **0.004**	614.9 (388.4; 951.8) 0.037	**0.0005**	0.203
Leptin (ng/ml)	0.7 (0.3; 1.3)	1.5 (1.2; 3.2) **0.021**	6.5 (3.5; 7.1)	1.6 (0.4; 3.8) **0.019**	0.7 (0.1; 3.2) **0.034**	2.3 (1.6; 4.0) 0.105	**0.0005**	0.728
Resistin (ng/ml)	36.4 (34.7; 38.4)	27.1 (25.2; 29.0) **0.0004**	20.0 (18.5; 22.8)	19.0 (17.8; 22.1) 0.517	21.9 (18.7; 22.6) 1.000	17.8 (15.4; 18.6) 0.163	**0.0002**	**0.002**
CRP (μg/ml)	23.9 (20.6; 26.6)	21.5 (18.3; 22.7) 0.105	30.8 (29.1; 32.6) n=8	30.7 (24.6; 33.0) 0.704	32.6 (24.9; 34.0) 0.916	29.8 (23.3; 30.9) 0.316	**0.001**	**0.026**
IL-6 (pg/ml)	1.9 (0.0; 1.9)	1.9 (1.9; 3.6) 0.057	3.6 (1.9; 5.2)	8.4 (3.6; 20.4) 0.122	16.0 (6.0; 24.8) **0.041**	3.6 (2.7; 5.6) 0.931	**0.018**	**0.023**
MMP-3 (ng/ml)	64.9 (54.6; 86.9)	73.9 (61.4; 91.9) 0.417	52.2 (38.0; 56.6)	52.9 (43.1; 64.6) 0.403	57.6 (46.9; 79.3) 0.299	47.6 (41.2; 52.9) 0.940	**0.044**	**0.018**

Serum samples were from adipsin-deficient (*Df^-/-^*) and wild type (*Df^+/+^*) 20-week- and 20-month-old mice. *Df^+/+^*(L) refers to 20-month-old *Df^+/+^* mice with a low Osteoarthritis Research Society International (OARSI) score (score, 2-4) while *Df^+/+^*(H) refers to those with a high OARSI score (score, 5-6). Data are expressed as the median (interquartile range). Differences between groups were assessed by the Mann-Whitney test. P values ≤0.050 were considered significant and are shown in bold. p^‡^ compares *Df^-/-^* and *Df^+/+^*mice; p^†^: 20-month-old *Df^-/-^* and *Df^+/+^*(L) mice; p^††^: 20-month-old *Df^-/-^* and *Df^+/+^*(H) mice; p*: 20-week- and 20-month-old *Df^-/-^* mice and p**: 20-week- and 20-month-old *Df^+/+^*mice. No statistical differences were found between *Df^+/+^*(L) and (H) mice.

Among the two inflammatory factors, only IL-6 showed a trend toward lower levels in the *Df*^-/-^ mice at both time points. No significant change was observed for levels of CRP and the matrix metalloproteinase (MMP)-3, when comparing *Df*^-/-^ with *Df*^+/+^ young and old mice. In the 20-month-old animals, comparison between *Df*^-/-^ and *Df*^+/+^(L) subgroup in the mice revealed similar changes, except for IL-6 where a significantly lower value was found.

Age-associated changes showed that the adiponectin level did not change, but in the older mice, levels of FGF-21 and leptin significantly increased in the *Df*^-/-^ mice, while resistin decreased in both *Df*^-/-^ and *Df*^+/+^ mice. The levels of the inflammatory factors CRP and IL-6 increased significantly with aging in both *Df*^-/-^ and *Df*^+/+^ mice, while those of the MMP-3 decreased significantly in the aging mice.

### Effect of in-vitro adipsin-silencing on adipokine expression

As Table 1 demonstrated that the in vivo silencing of adipsin in mice resulted in increased serum levels of FGF-21 and adiponectin in both young and aged mice, we further examined whether the lack of adipsin directly impacted FGF-21 expression in vitro. To that effect, siRNAs specific for the adipsin gene were transfected into the human hepatocyte cell line HepG2, as these cells are known to express both adipsin and FGF-21. Data revealed that silencing the adipsin gene expression with specific siRNAs (n=5) decreased adipsin expression (fold change±standard deviation, 0.77±0.07, p<0.0001) and this decrease resulted in a significant increase in expression of FGF-21 (3.85±2.95, p=0.043) after 24 hours. Adiponectin expression was also increased but did not reach statistical significance (11.77±13.99, p=0.133). Adipsin-silencing did not quite significantly affect the expression of another adipokine, leptin (1.62±1.39, p=0.060).

## DISCUSSION

Our study revealed that in spontaneous OA, the earliest sign of articular alteration appears to originate in the ACL and the lack of adipsin protects against the spontaneous development of OA. Data further showed that an increased production of the adipokines FGF-21 and adiponectin, and a decrease of the inflammatory factor IL-6 are possible mechanisms by which this protection could occur.

The data showing that adipsin plays an important role in articular tissues in OA are further reinforced by those from a recent publication in which adipsin-deficiency in an immunologically-induced inflammatory arthritis mouse model also demonstrates a protective role in articular tissues [42].

OA is now regarded globally as a whole joint disease, and our histological data of the wild-type mice (*Df*^+/+^) concur with this premise. Indeed, human primary OA is characterized by the degradation of cartilage and meniscus and alterations of the synovial membrane and subchondral bone [43, 44]. In this study, an important finding is that the ACL appears to be the earliest articular tissue to show signs of alterations during spontaneous age-related OA. This agrees with the notion that changes in the knee ligaments, and more particularly in the ACL, may precede cartilage degradation by inducing instability, consequently leading to articular tissue lesions and degeneration as observed in humans [38] and animals [39–41], and a recent publication reporting that an early ligamentous degeneration preceded the onset of human OA [45]. This finding also supports the use of the transection of the ACL as a preferred model to mimic the human disease. Indeed, in large animals (e.g. dogs), such a model was demonstrated to induce the disease with alterations strikingly similar to those of the natural disease in humans [46]. Moreover, the results obtained with two drugs/agents tested in the dog ACL model of OA (licofelone [47–49] and doxycycline [50]) were translated to human in clinical trials [51, 52].

The finding that adipsin-deficiency resulted in a significant increase of the serum levels of FGF-21 and adiponectin is interesting as both adipokines were reported to have metabolic/anti-inflammatory effects and suggested to be instrumental in maintaining joint health [53–57].

FGF-21 is an adipokine/endocrine hormone produced by the liver, adipose tissue and blood cells [58, 59], and is reported to be anti-inflammatory and to demonstrate anti-aging properties [60, 61]. Importantly and related to arthritis, FGF-21 was found to attenuate the collagen-induced arthritis by reducing, among other things, some pro-inflammatory cytokines [55]. The exact mechanisms behind more elevated levels of FGF-21 in adipsin-deficient (*Df*^-/-^) than in the wild-type (*Df*^-+/+^) mice remains to be determined, but data from the siRNA experiment suggest a direct event. Hence, the silencing of the adipsin gene expression resulted in a significantly increased expression of FGF-21. As human cells were used for this experiment, data also indicates that the relationship between adipsin and FGF-21/adiponectin is not specific to mouse cells, but could also be applied to human cells. Although a relationship between the expression of adipsin and FGF-21 has been reported [62], the regulatory factors at play still remain to be determined. FGF-21 is known to be regulated by a number of metabolic factors, nutrients, and oxidative stress [58, 63, 64], and involved in the regulation of glucose and lipid metabolism [65–68]. This could suggest that the lack of adipsin impacted these metabolic factors. However, this is to no avail, since, as previously reported, the serum levels of triglycerydes, cholesterol and free fatty acids in adipsin-deficient mice were similar to those of the wild-type [69].

Adiponectin could be found in the circulation in three forms, and depending on its form, could have pro- or anti-inflammatory properties [24, 70, 71]. As for FGF-21, this adipokine in the serum was found to be significantly elevated in the adipsin-deficient (*Df*^--/-^) compared to the wild-type (*Df*^-+/+^) mice. As FGF-21 was reported to be a regulator of adiponectin expression/production [66, 72], such an up-regulation could reflect that FGF-21 could be the trigger. Moreover, the beneficial effect of FGF-21 in the protection of the articulation against OA development/progression in the adipsin-deficient mice could also be due to the fact that FGF-21 inhibits IL-6 [55]; an inflammatory factor that, although it did not quite reach statistical significance, showed a trend toward a lower serum level in the *Df*^-/-^ than the *Df*^+/+^ mice in young mice, and was statistically different when compared to *Df*^+/+^(L) mice. In turn, the lower IL-6 serum levels in the adipsin-deficient mice could also result from the lack of the activation of the alternative complement, as adipsin is an important element of this pathway [73, 74].

Surprisingly, although in the younger mice the serum leptin level was significantly lower in adipsin-deficient animals, in the older mice, a higher leptin serum level was found. Although leptin was reported to be associated with cartilage degradation [23, 75–78], this still remains controversial since other works did not find such an association [26, 79, 80]. Although speculative, it is possible that in the *Df*^-/-^ mice, the putative catabolic effect due to the increased leptin levels could be rendered non-relevant compared to the beneficial effect of FGF-21, adiponectin, and the decreased level of IL-6. The factors responsible for the increased levels of leptin in the *Df*^-/-^ older mice are yet to be identified, but it could be hypothesized that the lack of adipsin could have generated a disturbance in the energy metabolism, triggering leptin expression.

Resistin is an adipokine reported to be involved in insulin resistance and inflammation [25, 81]. Increased synovial fluid resistin levels have been detected in OA patients [82]; however, by using a mouse model, such a resistin increase was found to immediately follow traumatic knee injury and decline later on, supporting its role in the early stages of trauma-induced OA [83]. This agrees with the lower serum levels found in our study in the old mice in both adipsin-deficient and wild-type mice.

Our data showed no significant difference in the MMP-3 serum levels between the wild-type and adipsin-deficient mice, but did show a decrease with aging in both groups. This was surprising, as MMP-3 expression has been found to be increased in individuals with OA lesions [84, 85]. However, literature about MMP-3 serum levels in relation to age is scarce, and in the few existing reports, conclusions are contradictory depending on whether they are from a human or mouse. Thus, in humans, MMP-3 serum levels increase with age [86, 87], whereas their expression and production in mice in the intervertebral disk showed age-related decreases [88], the latter concurring with our data.

Although possible explanations are stated above as to how adipsin could impact the articular tissues and some serum factors, further research needs to be done to identify the exact mechanism of action on each articular tissue, as well as to define the intimate molecular relationship of its effect on some serum factors.

Another important observation underlined in this study is the occurrence of very severe articular tissue degradation in about half of the wild-type (*Df*^+/+^) animals. Although such a severe event has been reported [89, 90], the exact reason for it remains to be determined. Notably, none of the adipsin-deficient mice displayed such extensive degradation, which strongly supports the lack of adipsin as protecting the articular tissues. Moreover, the analysis of the serum levels has yielded another valuable insight. Indeed, although μCT and histological/ immunohistological analyses demonstrated differences between the *Df*^+/+^(L) with (H) subgroups, no significant differences were found in the serum levels of the factors studied between these two subgroups. Thus, the severity of the age-related knee joint lesions seen at the local level in the *Df*^+/+^(H) mice is not reflected at a systemic level. These very severe articular tissue alterations in the *Df*^+/+^(H) mice, in addition to the fact that the *Df*^+/+^(L) mice demonstrated significant histological/ immunohistological differences when compared to the *Df*^-/-^ mice, strongly suggests that metabolic factors play a more significant role at the early (or less advanced) stages of the disease and that their serum levels would reach a plateau later on as OA progresses and mechanical alterations develop, superseding the metabolic factors [91]. In the μCt and subchondral bone, the fact that no differences were observed between the *Df*^-/-^ and *Df*^+/+^(L) mice but were found with *Df*^+/+^(H) also suggests that the bone/subchondral bone tissue is affected later in the spontaneous OA process.

In conclusion, although surgically-induced OA models develop articular alterations rapidly and reproducibly, such models are posttraumatic. In naturally occurring OA, various etiological factors join forces to bring about the structural and molecular changes. The mouse spontaneous development of OA is translational and better mimics human primary OA. By using this mouse model, data demonstrated that the earliest OA tissue manifestation occurs in the ACL. Moreover, the spontaneous OA development in the mice used in this study confirms the role of the adipokine, adipsin, as a player in OA progression. This work also identified the beneficial effect of another adipokine, FGF-21, against OA development/progression. Therefore, in addition to reaffirming the value of the adipsin as a potential biomarker for OA and target in therapeutic strategies, this study reveals the potential beneficial effect of FGF-21 against OA, and provides support for this factor as a new avenue for therapeutic approaches in primary OA.

## MATERIALS AND METHODS

### Adipsin-deficient mouse model

Homozygous adipsin-deficient (*Df*^-/-^) and wild type (*Df*^+/+^) mice were generated by interbreeding *Df*^+/+^ and homozygous *Df*^-/-^mice offspring from founder mating *Df*^+/-^ pairs kindly donated by the Dana Farber Cancer Institute (Boston, MA, USA) [69] as previously described [27]. The mice were maintained in accordance with the Canadian Council on Animal Care; the protocol was reviewed and approved by the Institutional Animal Care Committee of the University of Montreal Hospital Research Center. All mice were housed individually and were kept in a 12-hour light/dark cycle; food and water were available *ad libitum.* As described previously [27, 69, 92], the mutant mice have no apparent abnormality in their development and body weight compared to the wild type mice. Genotyping was carried out by polymerase chain reaction (PCR) with genomic DNA extracted from ear punch biopsy samples as described previously [27]. The mice were received and bred for this study over a 3-year period (about 4-7 generations) which rule out a potential genetic drift independent of adipsin deficiency. Mice of 20-week-old (*Df*^-/-^ n=11, *Df*^+/+^ n=13) and 20-month-old (*Df*^-/-^ n=13, *Df*^+/+^ n=13) were sacrificed by pentobarbital sodium. The right knee joints were dissected free of tissue and serum were taken.

### Histology/Histomorphometry

Articular samples were fixed in 4% paraformaldehyde, pH 7.4 for 16 hours at 4°C (Sigma-Aldrich, Oakville, ON, Canada), decalcified in 10% ethylenediaminetetraacetic acid (EDTA) pH 7.3 for 12 days (Wisent, St-Bruno, QC, Canada) and embedded in paraffin as described [27]. Sections (5 μm) were deparaffinised in xylene followed by a graded series of alcohol washes prior to staining and were stained with Safranin *O*-fast green (Sigma-Aldrich) as previously described [27].

The cartilage and synovial membrane alterations were determined according to the OARSI scoring method [37]. Of note, about half of the aging *Df*^+/+^ mice (n=6; 46%) had severe knee lesions with a high OARSI cartilage score (score, 5-6) and were subsequently named *Df*^+/+^(H); the other *Df*^+/+^mice with a lower OARSI score (score, 2-4) (n=7; 54%) were named *Df*^+/+^(L).

The subchondral bone histomorphometry was done on the medial compartment as described [93], using a Leitz Diaplan microscope linked to a personal computer to examine three non-consecutive sections. Measurements were recorded for the subchondral bone percentage of bone volume to the total volume (% BV/TV), trabecular thickness (μm) and plate thickness (μm). To this end, a box with a fixed width (1,000 μm) and variable length was created, with the upper limit at the calcified cartilage-subchondral bone junction and the lower limit at the subchondral bone-trabecular bone junction. The mean distance between the upper and lower limit and the trabecular thickness were calculated automatically by the BIOQUANT OSTEO software (Nashville, TN, USA).

The tartrate resistant acid phosphatase (TRAP) assay was performed to monitor osteoclast activation in the subchondral bone. TRAP detection was done on histological sections embedded in paraffin. Sections were first deparaffinized, then stained for enzyme activity, and processed as described previously [94]; counterstaining was done with 0.05% Fast Green (Sigma-Aldrich). TRAP-positive staining in the subchondral bone were quantitated with the BIOQUANT OSTEO software and data expressed as % of TRAP positive area over the total area.

The histopathological grading of the anterior and posterior horns of the menisci was done using the Kwok et al. scoring method [90], which takes into consideration the evaluation of the surface integrity, cellularity, Safranin-*O* staining distribution and intensity; with a maximum grading score of 18 (anterior horn) and 15 (posterior horn).

Assessment of anterior cruciate ligament (ACL) integrity was performed on 5 μm sagittal sections that contained the whole ligament length, excluding the attachment sides at both ends. The presence of proteoglycans was detected by staining the sections with Safranin-*O* [95]. Images were taken at 100X and the red staining (representing the proteoglycans) in the core portion of the ligament was quantified with the BIOQUANT OSTEO software and data expressed as % proteoglycans (red stained) area over total area.

The collagen organization in the ACL was evaluated on 5 μm paraffin sections following sirius red staining as described [96]. In brief, each slice was stained with a 0.1% sirius red solution and images at 100X were taken under polarized light. The dark background of the image was removed for further image processing with Adobe Photoshop software. The red (fibers structural component) and the green (altered fibers) areas were quantified separately with the BIOQUANT OSTEO software and data expressed as % of altered fibrils (green staining) area over the total area.

### Immunohistochemistry

Immunochemical analysis of the ACL was performed on 5 μm paraffin sections as described [27]. The tissues were successively incubated for 1 hour at 37°C with 1 mg/ml collagenase type I (USB, Cleveland, OH, USA) pH 7.4 in presence of 0.1% CaCl_2_, 1% hyaluronidase pH 6.0 in phosphate-buffered saline (PBS) and 1 mg/ml pepsin (all from Sigma-Aldrich) in 0.5M acetic acid. The tissues were then treated with 2% H_2_O_2_ (Fisher, Fair Lawn, NJ, USA) in PBS and with 1.5% goat serum (Vector Laboratories, Burlingame, CA, USA) in PBS for 15 and 45 minutes at room temperature, respectively. The primary antibody was an anti-human rabbit polyclonal antibody raised against type II collagen (dilution 1:200, Abcam, Cambridge, UK). Slides were incubated with Vectastain ABC kit (Vector Laboratories) according to the manufacturer’s specifications. The color was developed with 3,3′-diaminobenzidine containing hydrogen peroxide and nickel, and the slides were counterstained with eosin. Control procedures were performed according to the same experimental protocol as follows: (i) omission of the primary antibody, and (ii) substitution of the primary antibody with a non-specific immunoglobulin G (IgG) from the same host (rabbit) as the primary antibody (Santa Cruz Biotechnology, Dallas, TX, USA). Controls showed only background staining. Images were captured at 100X with a Leitz Diaplan microscope connected to the BIOQUANT OSTEO software. Surface area of the positive type II collagen ACL matrix staining was measured and data expressed as % of positive stained area over total area.

### Micro-computed tomography (μCT)

The μCT analysis was performed as described [97] on knee joints from 20-month-old *Df*^-/-^, *Df*^+/+^ (L) and *Df*^+/+^ (H) mice. Briefly, the knee joints were scanned using a Skyscan 1176 micro-CT scanner at 50KV and 500 μA, with a pixel size of 9 μm and a 0.5-mm aluminium filter. Data were recorded at every 3-degree rotation step through 180°. Image slices were reconstructed using NRecon software (version 1.6.3.2, Skyscan, Micro Photonics Inc., Allentown, PA, USA).

### Serum levels of adipokines/inflammatory factors/proteinases

Blood samples were obtained from 20-week-old and 20-month-old mice. The samples were allowed to coagulate and then centrifuged (4,000 rpm/1,400g, 10 minutes). The samples were stored at -80°C until analyzed. The levels of factors (adipokines, inflammatory factors, growth factors, MMPs) known to have a role in both aging and OA, whether beneficial or detrimental, were determined with specific assays (Luminex assay, R&D systems, Minneapolis, MN, USA) according to the manufacturer’s specifications, and quantitated using the Bio-Plex 200 apparatus (Bio-Rad, Mississauga, ON, Canada). An 8-point standard curve was used for each marker. Data were analyzed with the Bio-Plex Manager software (Bio-Rad).

The factors tested, as well as the minimum detectable doses and dilutions used were: adiponectin, 7.55 pg/ml (dilution 1:2000); CRP, 13.3 pg/ml (1:2000); FGF-21, 0.9 pg/ml (1:2); GM-CSF, 1.64 pg/ml (1:2); HGF, 3.33 pg/ml (1:2); IL-6, 2.3 pg/ml (1:2); IL-7, 35.4 pg/ml (1:2); IL-10, 8.2 pg/ml (1:2); IL-17, 7.08 pg/ml (1/2); leptin, 12.6 pg/ml (1:2); MCP-1, 134 pg/ml (1/2); MMP-3, 0.332 pg/ml (1/200); MMP-8, 2109 pg/ml (1:2); RAGE, 18.0 pg/ml (1:2); resistin 0.74 pg/ml (1:2000); S100A8, 61.3 pg/ml (1:2); S100A9, 3.55 pg/ml (1:2); TNF-α, 1.47 pg/ml (1:2); and VEGF, 3.96 pg/ml (1:2).

### Cell cultures

The human hepatocarcinoma cell line HepG2 was purchased from American Type Culture Collection (Manassas, VA, USA). The cells were routinely grown in low-glucose Dulbecco’s modified Eagle medium (DMEM) (Gibco, Thermo Fisher Technology, Waltham, MA, USA) supplemented with 10% heat-inactivated fetal calf serum (PAA Laboratories Inc, Etobicoke, ON, Canada) and an antibiotic mixture (100 units/ml penicillin base and 100 μg/ml streptomycin base; Wisent Inc, St-Bruno QC, Canada) at 37°C in a humidified atmosphere.

### Gene silencing

Gene silencing was basically done as described previously [98]. Briefly, a siRNA pool specific for the human adipsin gene was purchased from Thermo Fisher Technology. The siRNAs (final concentration 100 nM) were transfected for 24 hours with the Lipofectamine RNAiMax reagent (final concentration 0.2%, Thermo Fisher Technology) into HepG2 (200,000 cells/well, in 12-wells plates). Cells transfected with random non-targeting siRNAs (Ambion, Thermo Fisher Technology, Austin, TX, USA) served as controls.

### RNA extraction and real-time PCR

Total RNA from the transfected cells was extracted, quantified and reverse-transcribed as previously described [27]. Real-time PCR was done using the SYBR Green Master Mix (Qiagen, Valencia, CA, USA) [99].

The primers used in the PCR assays were 5-GCCT TGAAGCCGGGAGTTATT (S) and 5-GTGGAGCGA TCCATACAGGG (AS) (FGF-21); 5-TATGATGGCTC CACTGGTA (S) and 5- GAGCATAGCCTTGTCCT TCT (AS) (adiponectin); 5- CCTGCATCTGGTTGGTC TTT (S) and 5- CCTGCGTTCAAGTCATCCTC (AS) (adipsin); 5′- TCCCCTCTTGACCCATCTC(S) and 5′-GGGAACCTTGTTCTGGTCAT(AS) (leptin); 5- GG CAGCATCTACAACCCTGA (S) and 5- CCAGGA CTCGTTTGTACCCG (AS) (RPLPO, housekeeping gene).

The effect of siRNAs specific for adipsin on the expression of adipsin, FGF-21, adiponectin and leptin in the transfected HepG2 cells was calculated as fold change over the siRNA (random) controls which were assigned an arbitrary value of 1 and calculated as 2^-ΔΔCt^. Statistical analysis was done using the one-sample t-test, comparing the siRNA adipsin values (n=5 for each cell type) to the control random siRNAs values.

### Statistical analysis

Values are expressed as median (interquartile range) unless indicated. Statistical analyses were performed with the Fisher’s exact test or the Mann-Whitney U test (GraphPad Prism software, GraphPad, San Diego, CA, USA) where appropriate; a p≤0.050 was considered significant.
